# Chronic Hepatitis C Association with Diabetes Mellitus and Cardiovascular Risk in the Era of DAA Therapy

**DOI:** 10.1155/2018/6150861

**Published:** 2018-08-13

**Authors:** Sylvia Drazilova, Jakub Gazda, Martin Janicko, Peter Jarcuska

**Affiliations:** ^1^Department of Internal Medicine, Hospital Poprad, Poprad, Slovakia; ^2^1st Department of Internal Medicine, PJ Safarik University, Faculty of Medicine and L Pasteur University Hospital, Kosice, Slovakia

## Abstract

Patients with chronic hepatitis C have both higher prevalence of diabetes mellitus type 2 (T2DM) and increased cardiovascular risk compared to never infected people. Sustained viral response (SVR) achievement led to decreasing incidence and prevalence of T2DM during the interferon era of HCV treatment. Currently, direct-acting antiviral drugs (DAA) are the gold standard for treating HCV infection, while yielding SVR in nearly all patients. In chronic HCV patients with T2DM (prediabetes most likely too), DAA therapy is associated with both better fasting glucose and glycated hemoglobin (HbA1C) controls; thus reducing pharmacotherapy in a certain part of patients is possible. Papers mentioned in the review confirmed DAA role in both total cholesterol (TC) and low-density lipoprotein cholesterol (LDL-C) increase. This alteration was accompanied by an increase in high-density lipoprotein cholesterol (HDL-C) and a decrease in triglycerides (TG) verified by most of the studies. However, the clinical significance of lipoprotein alterations caused by DAA therapy has not been explained yet. Moreover, DAA treatment of chronic hepatitis C improves hypertension control and atherosclerotic plaques. It is very likely that DAA therapeutic regimens will decrease both T2DM prevalence and cardiovascular risk in chronic hepatitis C patients; further research, however, is needed.

## 1. Introduction

Chronic hepatitis C virus (HCV) infection affected some 170 million people worldwide in 2013 [1]. According to the latest information, in the view of better screening, diagnostics, and discovery of effective therapeutic regimens, the hepatitis C virus prevalence has been decreasing to currently 70 million people worldwide [2]. Chronic HCV infection tends to progress to liver fibrosis and cirrhosis. Subsequently, hepatocellular carcinoma can develop in the context of bridging fibrosis (F3 by Metavir) or liver cirrhosis (F4 by Metavir).

Decompensated liver cirrhosis together with hepatocellular carcinoma is the most common cause of death associated with chronic HCV infection [3].

Nowadays, chronic HCV infection is considered a systemic disease, while it does not affect only the liver, but other organs as well. Nearly three-quarters of patients also suffer from extrahepatic manifestations, which can already be seen before the diagnosis of chronic HCV infection [4]. Diabetes mellitus type 2 (T2DM) is one of the most common extrahepatic manifestations of chronic HCV infection [5]. Moreover, HCV accelerates atherogenesis and is also associated with cardiovascular disorders [6]. HCV infection increases not only liver disease mortality rate but also cardiovascular and all-cause mortality rate [7].

The primary goal of chronic HCV infection treatment is to achieve sustained viral response (SVR), characterized by the complete disappearance of hepatitis C virus from patient's body. SVR is associated with decreased liver disease mortality rate together with all-cause mortality rate [8]. Recently, there has been a remarkable breakthrough in the treatment of chronic HCV infection, in the form of the implementation of direct-acting antivirals (DAA) into clinical practice guidelines. Using a combination of at least two of DAA, specifically NS5A inhibitor, NS5B inhibitor, or NS3/4a protease inhibitor, results in a very high response rate [9].

This review aims to briefly describe the association between insulin resistance, T2DM, atherogenesis, and cardio-cerebrovascular disorders on one hand and chronic HCV infection on the other. We also present the impact of the DAA therapy on glycide and lipoprotein metabolism, together with possible implications of the DAA therapy on cardio- and cerebrovascular risk.

## 2. Chronic HCV Infection, Insulin Resistance, and T2DM

Chronic HCV infection can lead to increased insulin resistance as hepatitis C virus impairs the hepatocyte insulin signaling pathway in multiple ways [10], including (i) increased production of tumor necrosis factor-*α*, (ii) phosphorylation of the insulin receptors, (iii) the overexpression of the suppressor of cytokines (SOC-3), and (iv) induction of SOC-7 [11–13]. Even in HCV infected patients without metabolic syndrome, not only liver, but also whole-body insulin sensitivity is impaired. It is assumed that HCV infected liver produces mediators, which cause increased insulin resistance at extrahepatic sites, mainly in skeletal muscles.

Increased insulin resistance plays pivotal role in the development of T2DM in patients with chronic HCV infection [5, 14, 15]. In fact, insulin resistance is already present in patients with chronic HCV infection with low-grade fibrosis and its prevalence is significantly higher in infected than in healthy controls population. Also, its presence positively correlates with the grade of fibrosis and portal inflammation [16]. Furthermore, insulin resistance increases the prevalence of hepatocellular carcinoma and cardiovascular events in patients with chronic HCV infection [17].

Pathological background of T2DM is insulin resistance. T2DM is one of the civilization diseases and, recently, its prevalence has been showing an increasing tendency. Currently, 350 million people are suffering from this disease worldwide [1]. In industrialized countries, its prevalence is even higher with overall estimated prevalence some 9% in Europe [18].

T2DM is a disease with severe socioeconomic consequences and leads to decreased life expectancy, particularly when diagnosed early in life [19].

Prediabetes is approximately four times more frequent in patients with chronic HCV infection than in healthy controls population. Predisposing factors for prediabetes are both older age and higher ALT levels [20]. The assumption is that one-third of the patients with chronic HCV infection could have T2DM [21]. According to meta-analysis, patients infected with HCV are at higher risk for development of T2DM than noninfected patients (OR: 1.68; 95% CI: 1.15-2.45) [22]. Another meta-analysis showed similar results both in retrospective (adjusted OR: 1.68; 95% CI: 1.15-2.20) and prospective (adjusted HR: 1.67; 95% CI: 1.28–2.06) studies. Moreover, a group of patients with HCV/HIV coinfection has increased T2DM prevalence than a group of patients infected with HIV exclusively (OR: 1.82; 95% CI: 1.27–2.38) [23]. Among patients with hepatitis C, male gender (OR: 1.26, 95% CI: 1.03-1.54) and age over 40 years (OR: 7.39, 95% CI: 3.82-9.38) had higher prevalence of T2DM [22]. Furthermore, according to other study, higher BMI, F4 at the elastographic examination of the liver, duration of hepatitis C infection, response to previous therapy, and positive family history for T2DM together with insulin sensitivity can predict the development of T2DM [1]. Prevalence of chronic HCV infection is higher in patients with T2DM compared to nondiabetic patients [24]. The prevalence of T2DM in a group of chronic HCV infected patients with liver cirrhosis is higher than in both patients not suffering from liver disease (adjusted RR: 8.71; 95% CI: 1.28-59.46) and patients with liver cirrhosis of other causes (adjusted RR: 2.03; 95% CI: 1.54-2.67). Predictive factor for T2DM development in HCV positive patients with liver cirrhosis is albumin level less than 39 grams per liter [25]. In HCV patients, new onset diabetes predicts decompensation of liver cirrhosis (RR: 2.01; 95% CI: 1.07-3.79; p<0.001) [26]. Interestingly, the incidence of diabetic retinopathy in patients with liver cirrhosis is significantly lower in HCV positive than in HCV negative patients [27, 28]. T2DM is considered to be accelerating carcinogenesis in patients with chronic HCV infection. There is a higher prevalence of hepatocellular carcinoma in the group of HCV positive patients with T2DM than in the group of HCV positive patients without T2DM [29].

Eventually, achievement of SVR could lead to a drop in the prevalence of T2DM in patients with chronic HCV infection in the near future. Insulin resistance leads to a worse therapeutic response to insulin therapy in patients with chronic HCV infection [30]. There are four studies which evaluated the effect of SVR achievement on T2DM incidence during the era of interferon therapy. A retrospective study from Japan followed 2 842 patients with chronic HCV infection treated with interferon therapy. The average duration of the follow-up was 6.4 years. The cumulative prevalence of T2DM was 3.6% at five years, 8.0% at ten years, and 17.0% at 15 years. Predictive factors for T2DM development were advanced liver disease (HR:3.30; 95%CI: 2.06-5.28; p < 0.001), failure to achieve SVR after therapy (HR: 2.73; 95%CI:1.77-4.20; p < 0.001), baseline prediabetes (HR: 2.19; 95%CI: 1.43-3.37; p < 0.001), and age ≥ 50 years (HR: 2.10; 95%CI: 1.38-3.18; p < 0.001) [31].

A prospective study from Spain followed 1 059 patients with chronic HCV infection treated with interferon therapy. Insulin resistance was a negative predictive factor for SVR achievement. SVR achievement (OR: 0.44; 95%CI: 0.20-0.97; p=0.004) together with fibrosis stage (OR: 1.46; 95%CI: 1.06-2.01; p=0.02) was defined as independent risk factors for both development of impaired fasting glucose and T2DM by logistic regression analysis [32].

Another retrospective study from Spain followed 234 patients. All patients had chronic HCV infection, neither had liver cirrhosis, and all of them were treated with interferon therapy.

Those patients, who had been able to achieve SVR, were in lower risk for development of glucose abnormalities (HR: 0.48; 95%CI: 0.24-0.98, p = 0.04) [33]. Lastly, in a retrospective study from Italy, there was no association described between the achievement of SVR and lower risk of developing T2DM. Patients in this study had been followed for over eight years. However, one should take into consideration the limitation represented by a very low number of patients followed in this study [34].

New diabetes mellitus treatment options discovered recently could improve glycemic and metabolic profile and cardiovascular risk [35]. Furthermore, recent prospective study showed that SGLT2 inhibitor for NAFLD complicated by T2DM improved hepatocyte steatosis and liver fibrosis [36]. New antidiabetic drugs introduced into the clinical practice currently and in the future will improve glycemic control in T2DM patients with chronic hepatitis C.

## 3. Chronic Hepatitis C and Lipoprotein Metabolism

HCV assembly and secretion are closely associated with synthesis and secretion of lipoproteins. Entry of HCV particles into hepatocyte is dependent on lipoproteins with apolipoproteins playing the principal role. Thus, lipoprotein-HCV interaction directly affects infectivity of HCV particles [37]. HCVs are secreted from hepatocyte as highly infective lipoviral particles, which contain mainly apolipoprotein C and apolipoprotein E [38].

Therefore, lipoproteins play the crucial role in HCV infectivity.

Chronic HCV infection is associated with the presence of fatty liver disease [6]. Fatty liver is approximately five times more frequent in genotype 3a than in genotype 1. It is not associated with BMI values nor ferritin values; on the other hand, it is very closely associated with HCV viral load. Patients with HCV genotype 3a have significantly lower total cholesterol values (TC) than patients with genotype 1 [39]. This type of lipid accumulation in the liver is called viral steatosis. There is no increased insulin resistance with viral steatosis, it does not lead to progression of liver fibrosis, and it does not affect the impact of interferon therapy. Viral steatosis vanishes after achieving therapeutic response and appears back during relapse of chronic hepatitis [6, 39, 40]. The association between viral steatosis and atherogenesis acceleration has not been studied yet. On the other hand, metabolic steatosis in chronic HCV infection is associated with insulin resistance, accelerates atherogenesis, leads to liver fibrosis progression, and worsens interferon therapeutic response. Metabolic steatosis and viral steatosis most likely also increase the risk of hepatocellular carcinoma in patients with chronic HCV infection [6].

Typical laboratory abnormality in HCV infected patients is viral hypolipidemia. There are lower TC and LDL-C levels in patients with chronic HCV infection than in noninfected patients. However, HDL-C and triacylglycerides levels are roughly the same in both groups of patients. Though, after achieving viral response, viral hypolipidemia disappears [41].

Hyperlipidemia in patients with chronic HCV infections is rather rare. Out of 280 patients with chronic HCV infection and thalassemia, only one had higher LDL-C levels (0.4%), and 19 had higher levels of TG (7%) [42]. Egyptian study describes lower TC, LDL-C, and TG levels in patients with chronic HCV infection than in noninfected patients. In contrast, participants who cleared HCV infection had higher triglyceride levels compared with those never infected. The question that remains to be answered is whether higher TG level in patients with chronic HCV infection does or does not increase the chance for spontaneous HCV clearance [43, 44].

Infected patients do not have atherogenic dyslipidemia. This fact is the reason why the incidence of metabolic syndrome is, after sex, gender, and fibrosis stage adjustment, not higher in infected patients than in healthy controls [45]. In spite of that, patients with chronic HCV infection are at higher cardiovascular risk than noninfected patients [46, 47]. The reason of that could be explained by studying individual fractions of lipoproteins. Japanese authors found that patients with chronic HCV infection, genotype 1, and advanced fibrosis had higher levels of LDL-TG, HDL-TG, and small VLDL-TG. LDL-TG and small VLDL-TG are responsible for the acceleration of atherogenesis. It becomes one of the possible explanations for atherogenesis acceleration in patients with chronic HCV infection and advanced liver fibrosis, albeit further research on the association between altered lipoprotein metabolism and accelerated atherogenesis in chronic HCV patients is needed [48].

Although hyperlipidemia with chronic hepatitis C is a rare finding, patients could benefit from statin therapy, while they have several pleiotropic effects in hepatology [49]. The addition of statins to pegylated interferon and ribavirin therapy increases the chance of achieving SVR (OR: 2,02; 95%CI 1,38-1,94) [50]. Statins retard hepatic fibrogenesis, mainly through reducing of microthrombus formation in hepatic circulation [49]. In the HALT-C study, statins reduced risk of fibrosis progression in nonresponders to pegylated interferon and ribavirin therapy (HR: 0,32; 95% CI 0,10-0,99) [51]. Statin use was also associated with a reduced risk of liver cirrhosis development in a dose-dependent manner among patients with chronic HCV infection compared to patients not treated with statins [52]. There is a possibility that statins could reduce the risk of hepatocellular carcinoma development in patients with HCV hepatitis. ERCHIVES study described the ability of statins to reduce the incidence of hepatocellular carcinoma among patients with chronic HCV infection (aHR: 0,60, 95%CI 0,53-0,68). This effect was time-dependent, and Rosuvastatin and Fluvastatin showed the best efficacy [53]. After all, there is no knowledge about how statins do influence atherogenesis and cardiovascular risk in HCV patients, yet.

## 4. Chronic Hepatitis C and Cardiovascular Risk

Although the association of chronic HCV infection with higher cardiovascular risk has not been confirmed yet, the majority of the research papers consider higher cardiovascular risk as one of the extrahepatic manifestations of HCV infection. The higher cardiovascular risk in HCV infection has multifactorial pathogenesis. As shown above, HCV infected patients have higher prevalence and risk of development of T2DM and its association with accelerated atherogenesis is well known. Furthermore, chronic HCV infection also accelerates atherogenesis by direct pathological pathways: (i) chronic systemic inflammation, (ii) chronic endothelial damage, and (iii) direct infection of the arterial wall.

**Figure 1:**
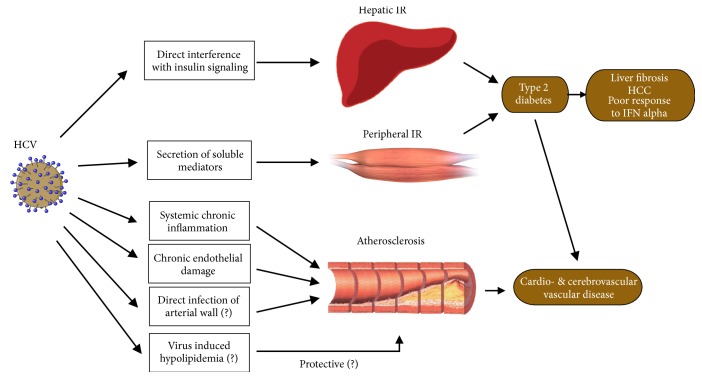
Schematic representation of interactions between hepatitis C virus and cardiovascular risk. Adapted from Negro 2014 [6].

T2DM together with accelerated atherogenesis increases cardio-cerebrovascular risk [6]. The question that we are still looking for the answer to is whether hypolipoproteinemia mentioned in the previous chapter could have a protective effect on atherogenesis (see Figure 1).

Atherosclerosis leads to both formation of atherosclerotic plaques in arteries and increased arterial intima-media thickness. Both carotid artery plaques and carotid intima-media thickness (cIMT) are subjects of studies. Chinese researchers published meta-analysis of eight studies. In seven of them, HCV infection significantly increased the risk of carotid atherosclerosis compared to those never infected (adjusted OR: 1.76, 95%CI: 1.20-2.32) [76]. Two other studies shed light on the association of HCV seropositivity with coronary atherosclerosis. In the Turkish paper, HCV seropositivity was an independent predictor for severity of coronary atherosclerosis (OR: 2.02; (95%CI: 1.58-2.58, p<0.001) [77]. On the other hand, in the research performed in Japan, prevalence of anti-HCV antibodies in patients without coronary artery disease was 2.8% and in patients with coronary artery disease only slightly higher, 3.4%, although limitation to this research was a low number of examined patients [78].

Angina pectoris, myocardial infarction, and stroke are some of the most frequent signs of atherosclerosis. In a meta-analysis of 34 studies, all of which followed patients with coronary artery disease, unstable angina pectoris, myocardial infarction, and stroke, patients with chronic HCV infection were in significantly higher risk for cardio-cerebrovascular disease than noninfected patients (OR: 1.43; 95%CI: 1.21 - 1.68). Meta-analysis of 22 studies found the substantially higher risk for coronary artery disease in patients with chronic hepatitis C than controls (OR: 1.38; 95%CI: 1.10-1.73) [46]. Studies we mentioned previously had proved that chronic HCV infection raises the risk of both subclinical and clinically apparent cardio-cerebrovascular disease.

## 5. DAA Therapy Effect on Glycemia and Glycated Hemoglobin

Studies performed during the era of interferon therapy of chronic hepatitis C revealed a significant drop in fasting glucose and glycated hemoglobin (HbA1C) levels when patients achieved SVR. This drop was not observed in patients with chronic hepatitis C relapse [79]. Majority of papers evaluating the effect of DAA on fasting glucose or HbA1C followed patients with both chronic HCV infection and diabetes mellitus. Post hoc analysis of 6 studies that followed patients in 3a stage of chronic hepatitis C, genotype 1, treated with 3D combo paritaprevir/ritonavir + dasabuvir + ombitasvir, revealed that 68.7% of all patients included in the study had normal glycemia levels, 25.4% of patients had prediabetes, and 5.9% of patients had diabetes mellitus. There was a significant drop in fasting glucose in the group of patients who received treatment compared to the group that received placebo. In overall, notable drop in fasting glucose was observed (–8.87 mg/dL by week 12; p < 0.0001). The most significant drop in fasting glucose was recorded in the group of patients with T2DM (–22.1 mg/dL by week 12; p < 0.0001), followed by still significant drop of fasting glucose in the group of patients with prediabetes (–5.78 mg/dL by week 12; p < 0.0001). On the contrary, there was slight, not significant increase of fasting glucose in the group of patients with normal baseline fasting glucose levels (1.34 mg/dL by week 12; p = 0.057) [54].

Further studies assessed decrease in fasting glucose or HbA1C during therapy or after the completion of DAA therapy in patients with chronic hepatitis C and T2DM. Two of them followed patients with genotype 4 exclusively [63, 64] and the third followed a group of patients, where the majority of patients were infected with genotype 4 [62]. All three above-mentioned studies dealt with Egyptian population. A Japanese study followed chronic hepatitis C patients with genotype 1b exclusively [55]; another study observed patients with genotype 1, exclusively [56]. The remaining studies followed patients regardless genotype, while genotype 1 dominated [57–61]. Different treatment options were studied. Morales et al. used the combination of pegylated interferon, sofosbuvir, and ribavirin [58]. All other studies employed interferon-free regimens. Meissner et al. used sofosbuvir and ribavirin [56], Egyptian study used sofosbuvir and daclatasvir [63], another Egyptian study used sofosbuvir and simeprevir [64], and Japanese study used sofosbuvir and ledipasvir [55]. In two studies, the patients were not treated uniformly, though always sofosbuvir was used [58, 62]. In three Italian studies patients were treated mostly with combinations based on sofosbuvir, only small part of them was treated with paritaprevir/ritonavir + dasabuvir + ombitasvir [59–61].

Research performed in the US followed 2 435 patients from National Veterans Affairs healthcare system. They were treated with sofosbuvir and simeprevir, sofosbuvir and ledipasvir, or the combination of paritaprevir/ritonavir + dasabuvir + ombitasvir. None of them received ribavirin [57].

Both fasting glucose and HbA1C dynamics were evaluated in four analyses [59, 61–63], two studies evaluated changes in fasting glucose solely [60, 64], and four studies reported HbA1C dynamics only [55–58]. All of them observed significant drop in fasting glucose levels or HbA1C during or after the completion of therapy [55–64].

New Zealand study followed patients after liver transplantation. Out of 91 treated patients, 62 were nonresponders on previous therapy. More than half of them were infected with hepatitis C virus genotype 1. Majority of patients received combination based on sofosbuvir; three patients received a combination of paritaprevir/ritonavir + dasabuvir + ombitasvir, and six patients were treated with glecaprevir + pibrentasvir. Out of all patients, 96% achieved SVR. HbA1C values dropped from 35.5±4.3mmol/mol to 33.3±3.6 mmol/mol at 44 weeks after treatment (p = 0.03). Those patients, who were not treated with antidiabetics, were observed with fasting glucose level drop from 6.8±1.7mmol/L before therapy to 5.7±1.1mmol/L 24 weeks after completion of therapy [65].

In contrast to that, a prospective study followed 251 patients with chronic HCV infection, genotype 1 a/b. Out of all patients, 31% were HIV positive, and 17% of patients had T2DM, out of whom 79% were treated with antidiabetic therapy. One patient was treated with pegylated interferon, ribavirin, and telaprevir; other patients were treated with various antivirotics, including sofosbuvir, ledipasvir, beclabuvir, daclatasvir, and asunaprevir. Contrary to the previous study, after completion of therapy, HbA1C levels did not differ in patients who achieved SVR from those patients who failed to achieve SVR. HbA1C drop in patients with SVR was 0.022±0.53% (NS). Also, changes in HbA1C levels after completion of therapy were roughly the same in the group of HCV/HIV coinfected patients with SVR and in the group of HCV/HIV coinfected patients without SVR. Moreover, HbA1C levels did not differ between a group of diabetic patients with SVR and group of diabetic patients without SVR after completion of therapy. The limitation of this study was a low number of patients with T2DM comorbidity, and different DAA regimens used [66]. The results of the studies mentioned above are summarized in Table 1.

The drop in fasting glucose was not observed in all patients. Italian study found a drop in fasting glucose levels in 67% patients and a drop in HbA1C in 80% of patients with chronic hepatitis C and T2DM [61]. Egyptian paper analyzed patients with chronic hepatitis C, genotype 4, and T2DM. Every patient achieved SVR. Drop in glycemia levels was observed in 77.2% patients 12 weeks after therapy. Prognostic factors for a drop of glycemia levels >20 mg/dl or a drop of HbA1C levels > 0,5% were identified in multiple logistic regression analysis. Prognostic factors were the duration of T2DM < 7 years, negative family history for T2DM, or any Child-Pugh A stage of liver disease [63]. Analysis based on National Veterans Affairs healthcare system database followed patients with both chronic hepatitis C and T2DM. The conclusion of this analysis describes a significant drop in HbA1C in patients who achieved SVR compared to patients with failure to achieve SVR [57].

One of the severe complications of T2DM during DAA therapy is hypoglycemia. Spanish authors published case report of a well-compensated diabetic patient before sofosbuvir + ledipasvir therapy. The patient received 18 units of basal insulin daily and every six to eight hours 4-8 units of bolus insulin based on glycemia. HbA1C was 6.5%. From the 7th day of therapy on, his bolus insulin dose was reduced. Despite that, on the 18th day of the therapy, the patient presented with symptomatic hypoglycemia with glucose level 50 mg/dL. On the 21st day of the therapy, bolus insulin was discontinued, and later on, also basal insulin was discontinued. The decreased demand for insulin came with ALT normalization and HCV RNA disappearance from the patient's serum [80]. Thus, 3-40% of patients treated with antidiabetics are in a need of dose reduction during DAA therapy, while patients treated with insulin need dose reduction even more frequently [55, 57, 59, 61, 63, 65, 66].

All of the previously mentioned findings confirmed better compensation of diabetes mellitus in patients who are treated with DAA. Majority of studies included patients treated with therapeutic regimens based on sofosbuvir. Reducing antidiabetic therapy in a certain part of patients is possible.

## 6. The Effect of DAA on the Lipoprotein Metabolism

HCV life cycle requires lipoprotein particles. Therefore, alteration of lipoprotein profile after SVR achievement is possible. Austrian scientists described a significant increase in TC after SVR achievement in patients with genotype 3a. In contrast to that, patients with genotype 3a with failure to achieve SVR did not present with increase in TC values [39]. There are several papers concerning the effect of DAA on the lipoprotein metabolism. Three studies followed patients with genotype 1b exclusively [68–70], five studies dealt with patients with genotype 1 exclusively [56, 66, 67, 71, 72], and another five studies followed mostly genotype 1 [58, 65, 73–75].

Egyptian study followed patients particularly with genotype 4 [64]. One study dealt with patients after liver transplantation exclusively and another was concerned with coinfected patients [72]. Three studies dealt also with patients treated with interferon [58, 73, 75]. One study used combination of sofosbuvir + ribavirin [56], and another used combination of sofosbuvir + simeprevir [64]. Combination of daclatasvir + asunaprevir was applied also [68]. Two studies treated patients either with sofosbuvir + ledipasir or daclatasvir + asunaprevir [69, 71], and one research applied combinations of either sofosbuvir + ledipasir, daclatasvir + asunaprevir, or sofosbuvir + ribavirin [70]. Furthermore, combinations of sofosbuvir + ledipasir or grazoprevir + elbasvir were applied [67], one study used only sofosbuvir based therapy [58], and four studies treated patients mostly with sofosbuvir based combinations [65, 72–74]. Moreover, two studies used different combinations of DAA [66, 75].

Conclusions of above-mentioned studies were as follows. One study observed an increase in TC (LDL-C was not assessed) [67], and another observed an increase in LDL-C (TC was not assessed) [56]. In the 12 remaining studies increase of both TC and LDL-C was recorded [58, 64–66, 68–75]. Three studies described a significantly higher increase in both TC and LDL-C when the combination of sofosbuvir + ledipasvir was used compared to the combination of daclatasvir + asunaprevir [68–70]. Some of the studies mentioned above assessed HDL-C dynamics also. Four papers described an increase in HDL-C during or after treatment [64, 68, 69, 72], and two papers observed no alteration in HDL-C [65, 73]. Furthermore, seven studies assessed TG during and after treatment. Four papers described a decrease in TG [56, 66, 67, 70], although three studies observed no changes in TG [65, 72, 73]. One study dealt with TG dynamics during the therapy with the combination of paritaprevir/ritonavir + dasabuvir + ombitasvir. There was a significant drop in TG compared to the group of patients who received placebo. The most significant drop was observed in the group of patients who had presented with hypertriglyceridemia before the therapy. In contrast to that, there was a small, but still significant, increase in hypertriglyceridemia in the group of patients presenting with normal triglycerides levels before the therapy [54]. The studies on the lipoprotein metabolism are summarized in Table 2. Japanese authors described both significant increase in lipoprotein(a) and alteration of apolipoprotein B/apolipoprotein A1 ratio, in chronic hepatitis C patients with genotype 1 after the completion of DAA treatment [74]. A study from the US observed a significant drop in both apolipoprotein AII and apolipoprotein E and a significant increase in apolipoprotein C in chronic hepatitis CII patients with genotype 1 after DAA treatment [81].

Considering all of the studies mentioned above, the effect of DAA therapy on atherogenesis after achieving SVR is hard to assess. There is a significant increase in both TC and LDL-C on one side and a considerable increase of HDL-C together with a considerable decrease of TG, on the other. Further research, with a high number of patients along with lipoprotein fractions and subfractions dynamics assessment, is needed regarding the effect of lipoprotein metabolism alterations on atherogenesis.

Importantly, besides the effect on glycemia and lipoprotein metabolism, DAA treatment affects atherogenesis by other means as well. According to the latest information, SVR achievement after DAA therapy improves carotid atherosclerosis directly [82]. Moreover, New Zealand study observed blood pressure improvement in patients after liver transplantation [65].

## 7. Conclusion

Chronic hepatitis C is associated with both the development of insulin resistance and T2DM. In spite of viral hypolipidemia, infected patients are at higher cardiovascular risk. The positive effect of SVR achievement on decreasing incidence and prevalence of T2DM was proved already during the interferon era of HCV treatment. DAA therapy of chronic HCV infection is yielding SVR in nearly all patients. However, more epidemiological research is needed regarding the effect of SVR achievement on the development of T2DM. More importantly, DAA therapy leads to both better fasting glucose and HbA1C controls in patients with T2DM, and with prediabetes most likely also. Reducing antidiabetic treatment in some of the patients is possible. According to conclusions of the preliminary studies, DAA therapy improves hypertension control and atherosclerotic plaques. Furthermore, DAA therapy alternates lipoprotein profile considerably. Further research, however, is needed to evaluate its clinical significance. Most likely, DAA treatment and subsequently SVR achievement decrease cardiovascular risk. This fact is another reason for early treatment of patients, including those with a lower grade of liver fibrosis. Yet, chronic hepatitis C treatment remains inaccessible not only in developing countries but also in countries with high quality of life [83].

## Figures and Tables

**Table 1:** Studies reporting the changes of fasting glucose, HbA1C, and antidiabetic treatment after DAA treatment.

Author	Country	Patients	Genotype	Treatment	Decrease of the fasting glucose during or after DAA treatment	Decrease of the HbA1C during or after DAA treatment	Proportion of patients with the reduction of antidiabetic treatment
Tran,2017 [54]	Multi- ethnic	General HCV population, 25.4% prediabetes 5.9% T2DM	Genotype 1	Paritaprevir/ ritonavir + dasabuvir + ombitasvir	Yes, in all patients, patients with prediabetes and T2DM	NA	NA

Ikeda, 2017 [55]	Japan	T2DM	Genotype 1b	Sofosbuvir + ledipasvir	NA	Yes	NA

Meissner, 2015 [56]	USA	T2DM	Genotype 1	Sofosbuvir + ribavirin	NA	Yes	NA

Hum [57]	USA	T2DM	Mostly genotype 1	Sofosbuvir + simeprevir or Sofosbuvir + ledipasvir or Paritaprevit/ritonavir + dasabuvir + ombitasvir	NA	Yes	9%

Morales, 2016 [58]	USA	T2DM	Mostly genotype 1	Only sofosbuvir based	NA	Yes	25%

Ciancio, 2018 [59]	Italy	T2DM	Mostly genotype 1	Mostly sofosbuvir based	Yes	Yes	21%

Fabrizio, 2017 [60]	Italy	T2DM	Mostly genotype 1	Mostly sofosbuvir based	Yes	NA	NA

Pavone, 2016 [61]	Italy	T2DM	Mostly genotype 1	Mostly sofosbuvir based	Yes	Yes	23%

Abdel Alem, 2017 [62]	Egypt	T2DM	Mostly genotype 4	Only sofosbuvir based	Yes	Yes	NA

Dawood, 2017 [63]	Egypt	T2DM	Genotype 4	Sofosbuvir + daclatasvir	Yes	Yes	27%

El Sagher, 2018 [64]	Egypt	T2DM	Genotype 4	Sofosbuvir + simeprevir	Yes	NA	NA

Beig, 2018 [65]	New Zealand	LTx patients, only patients without antidiabetic treatment	Mostly genotype 1	Mostly sofosbuvir based	yes	Yes	40%

Chaudhury, 2017 [66]	USA	General population, 31% HIV positive, 17%T2DM	Genotype 1	Multiple DAA	NA	No	3%

NA: not available; T2DM: type 2 diabetes mellitus.

**Table 2:** Studies reporting the changes of lipoprotein metabolism after DAA treatment.

Author	Country	Genotype	Treatment	Increase of total cholesterol during or after DAA treatment	Increase of LDL-C during or after DAA treatment	Increase of HDL- C during or after DAA treatment	Decrease of TG during or after DAA treatment
Sun [67]	Taiwan	Genotype 1	Sofosbuvir + ledipasvir or Grazoprevir + elbasvir	Yes	NA	NA	Yes

Meissner [56]	USA	Genotype 1	Sofosbuvir + ribavirin	NA	Yes	NA	Yes

Chida [68]	Japan	Genotype 1b	Daclatasvir + asunaprevir	Yes	Yes	Yes	NA

Endo [69]	Japan	Genotype 1b	Sofosbuvir + ledipasvir or daclatasvir + asunaprevir	Yes	Yes	Yes	NA

Inoue [70]	Japan	Genotype 1b	Sofosbuvir + ledipasvir or Sofosbuvir + ribavirin or daclatasvir + asunaprevir	Yes	Yes	NA	Yes

Chaudhury [66]	USA	Genotype 1	Multiple DAA	Yes	Yes	NA	Yes

Hashimoto [71]	Japan	Genotype 1	Sofosbuvir + ledipasvir or daclatasvir + asunaprevir	NA	Yes	NA	NA

Meissner [56]	USA	Genotype 1	Sofosbuvir + ribavirin	Yes	Yes	NA	NA

Townsend [72]	USA	Genotype 1	Mostly sofosbuvir based	Yes	Yes	Yes	No

Beig [65]	New Zealand	Mostly genotype 1	Mostly sofosbuvir based	Yes	Yes	No	No

Carvalho [73]	Portugal	Mostly genotype 1	Mostly sofosbuvir based	Yes	Yes	No	No

Gitto [74]	Italy	Mostly genotype 1	Mostly sofosbuvir based	Yes	Yes	NA	NA

Mauss [75]	Germany	Mostly genotype 1	Multiple DAA	Yes	Yes	NA	NA

Morales [58]	USA	Mostly genotype 1	Only sofosbuvir based	Yes	Yes	NA	NA

El Sagher [64]	Egypt	Genotype 4	Sofosbuvir + simeprevir	Yes	Yes	Yes	NA

Tran [54]	Multi- ethnic	Genotype 1	Paritaprevir/ ritonavir + dasabuvir + ombitasvir	NA	NA	NA	Yes, also in patients with baseline elevated TG

NA: not available, LDL-c: low density lipoproteins, HDL-c: high density lipoproteins, and TG: triglycerides.
